# Omentin Is Independently Associated with Stroke Severity and Ipsilateral Carotid Artery Stenosis in Patients with Acute Cerebral Ischemia

**DOI:** 10.3390/jcm10245797

**Published:** 2021-12-11

**Authors:** Maria Chondrogianni, Vaia Lambadiari, Aristeidis H. Katsanos, Maria Ioanna Stefanou, Lina Palaiodimou, Alexandros Stavros Triantafyllou, Georgios Karagiannis, Vasileios Konstantakos, Michael Ioakeimidis, Sokratis Triantafyllou, Christina Zompola, Chryssa Liantinioti, Alexandra Pappa, Ioannis Rizos, Konstantinos Voumvourakis, Georgios Tsivgoulis, Eleni Boutati

**Affiliations:** 1 Second Department of Neurology, “Attikon” University Hospital, School of Medicine, National and Kapodistrian University of Athens, 124 62 Athens, Greece; mariachondrogianni@hotmail.gr (M.C.); ar.katsanos@gmail.com (A.H.K.); marianna421@hotmail.co.uk (M.I.S.); lina_palaiodimou@yahoo.gr (L.P.); alexandros.st.triantafyllou@gmail.com (A.S.T.); geokarayian@hotmail.com (G.K.); va.konstantakos@gmail.com (V.K.); michael_ioakeimidis@hotmail.com (M.I.); socrates_tr@hotmail.com (S.T.); chriszompola@yahoo.gr (C.Z.); chrissa21@hotmail.com (C.L.); papaalek@gmail.com (A.P.); cvoumvou@outlook.com (K.V.); 2 Second Department of Internal Medicine, “Attikon” University Hospital, School of Medicine, National and Kapodistrian University of Athens, 124 62 Athens, Greece; vlambad@otenet.gr (V.L.); boutati@otenet.gr (E.B.); 3 Division of Neurology, McMaster University and Population Health Research Institute, Hamilton, ON L8S 3L8, Canada; 4 Second Department of Cardiology, “Attikon” University Hospital, School of Medicine, National and Kapodistrian University of Athens, 124 62 Athens, Greece; ioannis.c.rizos@otenet.gr; 5 Department of Neurology, University of Tennessee Health Science Center, Memphis, TN 38163, USA

**Keywords:** stroke, omentin, vaspin, acute cerebral ischemia, adipokines, carotid artery disease

## Abstract

Mounting evidence indicates an association between adipokines and inflammation-related atherosclerosis. Here, we sought to investigate the association of vaspin and omentin with clinical characteristics and outcomes of patients with acute cerebral ischemia (ACI). Consecutive ACI patients were evaluated within 24 h from symptom-onset. Stroke aetiology was classified using TOAST criteria. Adipokines were assayed using quantikine enzyme immunoassay commercially available kits. Stroke severity was assessed by NIHSS-score, and ipsilateral carotid stenosis (≥50% by NASCET criteria) by ultrasound and CT/MR angiography. Major cerebrovascular events were assessed at three months. We included 135 ACI patients (05 (78%) and 30 (22%) with acute ischemic stroke and transient ischemic attack, respectively; mean age ± SD: 59 ± 10 years; 68% men; median NIHSS-score: 3 (IQR:1–7)). Omentin was strongly correlated to admission stroke severity (Spearman rho coefficient: +0.303; *p* < 0.001). Patients with ipsilateral carotid stenosis had higher omentin levels compared to patients without stenosis (13.3 ± 8.9 ng/mL vs. 9.5 ± 5.5 ng/mL, *p* = 0.014). Increasing omentin levels were independently associated with higher stroke severity (linear regression coefficient = 0.290; 95%CI: 0.063–0.516; *p* = 0.002) and ipsilateral carotid stenosis (linear regression coefficient = 3.411; 95%CI: 0.194–6.628; *p* = 0.038). No association of vaspin with clinical characteristics and outcomes was found. Circulating omentin may represent a biomarker for the presence of atherosclerotic plaque, associated with higher stroke severity in ACI patients.

## 1. Introduction

Increasing attention has been drawn recently towards the establishment of blood-based biomarkers for acute ischemic stroke (AIS) and their incorporation into diagnostic and prognostic AIS models [1,2]. The interpretation of most available biomarkers for AIS, however, is currently limited by the high variability in reference ranges and confounders associated with underlying stroke aetiologies [1,3]. Adipokines, the cytokines secreted by adipose tissue, have emerged as promising biomarkers for cardiovascular and cerebrovascular disease, with mounting evidence supporting their role in atherosclerotic and vascular remodelling processes via pleiotropic actions on endothelial and smooth muscle cells [4,5]. Circulating adipokines have been well-associated with insulin resistance and vascular risk factors, including obesity, diabetes mellitus, gestational diabetes, hypertension and metabolic syndrome [6,7,8], yet their role in AIS remains, to date, poorly characterized.

Among the novel adipokines, omentin and vaspin have been proposed as candidate biomarkers for the prediction of stroke severity and functional outcome in AIS patients [7,9,10]. Omentin is considered an anti-inflammatory adipokine, which is mainly derived from the omental adipose tissue and has two highly homologous isoforms, omentin-1 and omentin-2 [9]. Omentin-1, which is hereafter referred to as omentin, comprises the main circulating isoform in human blood. Vaspin is a visceral adipose tissue-derived serine protease inhibitor with insulin-sensitizing effects [11], which, like omentin, is involved in processes of vascular inflammation, oxidative stress, atheroma formation, plaque ulceration and rupture [7,12,13].

Although significant associations between circulating omentin and vaspin levels and inflammation-related atherosclerosis have been previously reported, studies in AIS patients have yielded conflicting results on observed associations. Some investigators reported a negative association between circulating omentin levels and the risk of AIS, AIS severity and functional outcome following AIS [7,9,14], while other researchers reported positive associations [15]. Similarly, contradictory findings have been reported in studies assessing the association between circulating vaspin levels and AIS severity and prognosis [10,16].

Animal research has previously shown that circulating adipokines exhibit time-dependent responses to acute ischemic and inflammatory stimuli [17]. Fluctuations of adipokine levels after acute cerebral ischemia (ACI) may, thus, account for some disparate findings of earlier studies that recruited patients at varying time points, often with delays of several days from ACI onset [4,7,14]. As the timing of measurement has been acknowledged as a key factor when interpreting results of biomarker research [3,18], the potential diagnostic and prognostic yield of adipokines during the early phase of stroke remains to be established.

In view of the former considerations, we sought to investigate potential associations of circulating omentin and vaspin levels during the early phase of ACI with clinical and neuroimaging characteristics and outcomes of patients presenting within 24 h from ACI onset.

## 2. Materials and Methods

The data that support the findings of the present study are available from the corresponding author on reasonable request. This study was performed in accordance with the STROBE guidelines for reporting observational research [19].

### 2.1. Study Design and Regulations

We performed a single-centre, prospective cross-sectional study with blinded outcome assessment. Institutional review board approval was obtained prior to all study-related activity from the ethics committee of “Attikon” University Hospital (615 2/2/2015). Written informed consent was obtained from all patients or their legal representatives before enrolment.

### 2.2. Setting and Eligibility Criteria

The study was conducted at the Department of Neurology of “Attikon” University Hospital, Athens, Greece, a tertiary care facility covering the Western part of the Attica region. We prospectively enrolled consecutive adult (≥18 years) patients who presented to the emergency department with AIS or transient ischemic attack (TIA) during a 2-year period (January 2018–December 2019). Patients were excluded if the time elapsed from symptom onset exceeded 24 h, if they were diagnosed with intracranial haemorrhage or if they underwent acute neurosurgical procedures, including surgical decompression or external ventricular drain (EVD) placement. Patients with neoplastic disease, acute or chronic renal failure, hepatic failure, current infection, inflammatory disorders, sepsis or pregnancy were also excluded to rule out confounders that could interfere with measurements [7,20]. Finally, we excluded patients that failed to provide informed consent.

### 2.3. Data Collection

Data on demographic and clinical characteristics, including cardiovascular risk factors, were collected as follows: (1) demographic characteristics (age, sex); (2) vascular risk factors (atrial fibrillation, diabetes mellitus, hypertension, dyslipidaemia, congestive heart failure, history of stroke/TIA, history of myocardial infarction, current smoking, history of alcohol misuse, history of peripheral artery disease); (3) NIHSS-score (National Institute of Health Stroke Scale) on admission (NIHSS_adm_) and at hospital discharge (NIHSS_dis_); and (4) early neurological deterioration (END), defined as clinical worsening during the first 24 h after the qualifying event. Details regarding definitions of demographic and clinical characteristics have been previously published [21,22,23].

All patients underwent comprehensive diagnostic work-up during hospitalization in accordance with the American Heart Association (AHA) recommendations [24], including brain computed tomography (CT) or magnetic resonance imaging (MRI) scan with CT or MR angiography of cervical and cerebral vessels; ultrasonography of the carotid and vertebral arteries performed by vascular neurologists accredited in neurosonology; transthoracic or transoesophageal echocardiogram, a 12-lead electrocardiogram (ECG), or ECG Holter monitoring (>24 h); and routine blood tests/laboratory investigations, as standard of care.

All patients were classified according to discharge diagnosis as AIS or TIA, and classification of ischemic stroke aetiology was made according to the TOAST (Trial of ORG 10172 in Acute Stroke Treatment) criteria [25]. For sonographic assessment of carotid artery stenosis, the North American Symptomatic Carotid Endarterectomy Trial (NASCET) criteria were used [26], and vertebral artery stenosis ≥ 50% was deemed significant according to previously described sonographic criteria [27]. Additional details regarding classification of acute ischemic stroke patients and classification of carotid stenosis have been previously published [21,22,23]. All patients were prospectively followed-up, and the following major cerebrovascular events 90 days after the index event were captured: (1) death within 90 days and (2) recurrent stroke, defined as new AIS or TIA leading to hospitalization. All outcome events were assessed by attending-level stroke neurologists.

### 2.4. Sample Collection and Measurements

Venous blood samples were collected from all participating patients at prespecified time points within 24 h of admission and at 8:00 a.m. after at least 8 h of fasting. After at least 30 min of clotting, samples were centrifuged at 2500× *g* for 15 min, and all specimens were stored at −80 °C until testing. Omentin and vaspin are known to remain stable in blood samples frozen at −80 °C after several cycles of freezing and thawing [7].

Omentin was measured by a commercially available kit (Millipore Corporation, Billerica, MA, USA) with sensitivity 0.23 ng/mL, specificity 100%, intra-assay variation < 2.18%, interassay variation < 11% and lowest detectable level 0.23 ng/mL. Vaspin plasma levels were assayed by an ELISA kit (Adipogen Life Sciences, Liestal, Switzerland) with sensitivity 12 pg/mL, specificity 100%, intra-assay variation < 3.8% and interassay variation < 9%. All measurements were performed after full calibration of the assays according to manufacturers’ instructions by thoroughly trained external investigators and technicians. Our collaborative group has previously published details regarding vaspin and omentin measurements in patients with acute ischemic stroke and carotid artery disease [20,28].

### 2.5. Blinding

All study investigators performing diagnostic or clinical assessments were blinded to the results of laboratory tests and assays. External investigators and technicians conducting laboratory measurements were blinded to patient clinical and imaging data.

### 2.6. Statistical Analysis

We presented continuous parametric data using their mean values together with their corresponding standard deviations (SDs). We used median values with their corresponding interquartile ranges (IQR) for the presentation of nonparametric data and percentages for all dichotomous variables. Statistical comparisons between different subgroups were performed using the unpaired *t*-test and Kruskal–Wallis test adjusted for ties, as appropriate.

We used simple and multiple linear regression analyses to evaluate associations between omentin and vaspin levels with baseline characteristics, diagnostic parameters and clinical outcomes at discharge and at 90 days following the index event among patients with AIS or TIA. In all simple linear regression analyses, a threshold of *p* < 0.1 was used to identify candidate variables for inclusion in the multiple linear regression models that tested statistical significance hypothesis using the likelihood ratio test with an alpha value of 0.05. We reported all associations in linear regression models using unstandardized linear regression coefficients (LRC), with their corresponding 95% confidence intervals (95%CI). In a confirmatory analysis, Spearman’s rank–order correlation was run to assess the relationship between variables identified by the multiple regression analyses and circulating omentin and vaspin levels. The Stata Statistical Software Release 13 for Windows (StataCorp LP, College Station, TX, USA) was used for all statistical analyses.

## 3. Results

During the study period, 182 consecutively admitted patients were screened for eligibility, of whom 47 were excluded based on the predefined exclusion criteria (time from symptom onset > 24 h:21, intracranial haemorrhage:8, neoplastic disease:3, renal or hepatic failure:5, current infection or sepsis:8 and pregnancy:2). We included 135 consecutive ACI patients admitted within 24 h from symptom onset: 105 (78%) and 30 (22%) AIS and TIA patients, respectively. Baseline characteristics of the study population are presented in Table 1. The mean age (±SD) of included patients was 59 ± 10 years, 92 (68%) were men, and the median NIHSS-score on admission was 3 points (IQR:1–7). Concomitant cardiovascular risk factors, underlying aetiologies of the qualifying event in accordance with the TOAST classification and diagnostic results of the diagnostic work-up during hospitalization are summarized in Table 1. Among ACI patients, the mean (±SD) concentration of serum omentin was 10.2 ± 6.3 ng/mL, while the mean (±SD) measured serum vaspin concentration was 146.6 ± 177.1 ng/mL. There were no statistically significant differences in circulating omentin (*p* = 0.110) and vaspin (*p* = 0.682) levels between different types of underlying ACI aetiologies according to the TOAST classification.

**Figure 1:**
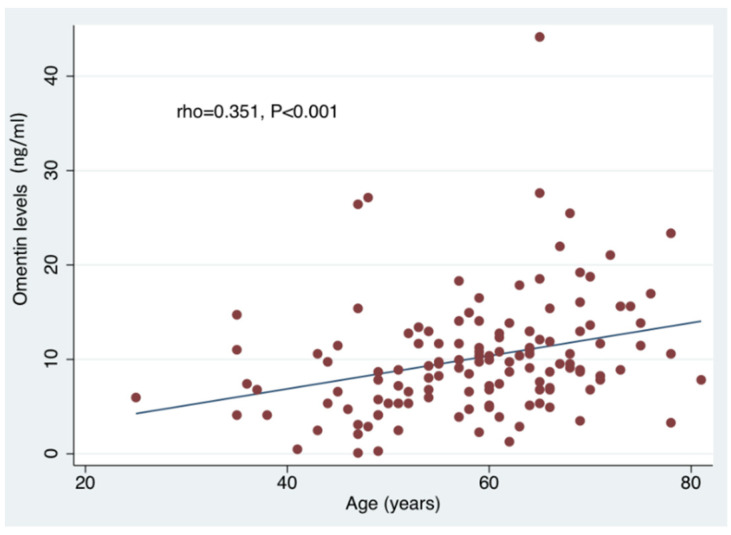
Spearman’s rank–order correlation depicts a significant correlation between age and circulating omentin levels (ng/mL).

**Figure 2:**
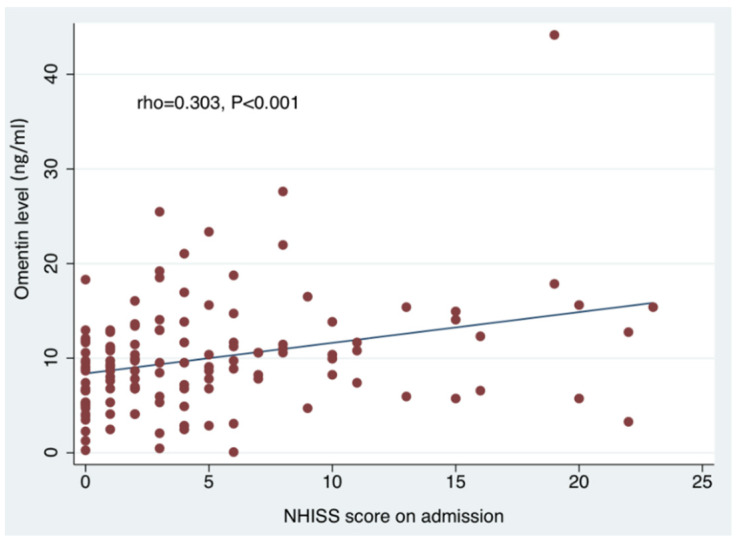
Spearman’s rank–order correlation depicts a significant correlation between NIHSS on admission and circulating omentin levels (ng/mL). NIHSS: National Institutes of Health Stroke Scale.

**Figure 3:**
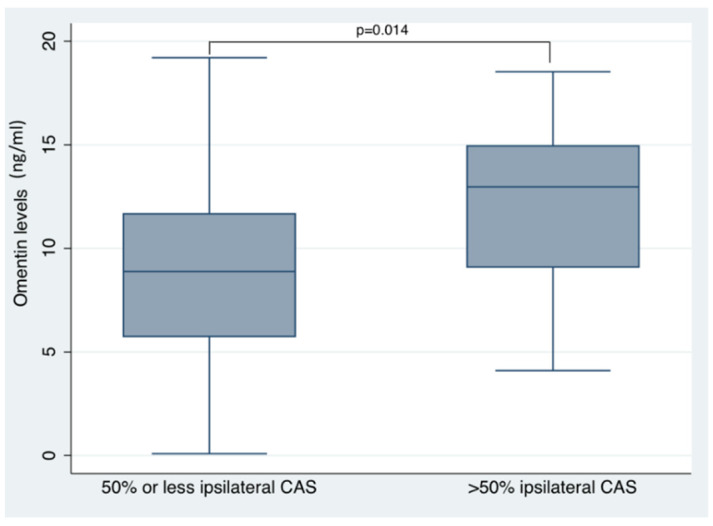
Significantly higher circulating omentin circulations are noted in patients with ≥50% ipsilateral carotid artery stenosis compared to patients without evidence of carotid artery stenosis. Within each boxplot, horizontal blue lines denote median values; lower and upper box boundaries correspond to the 25th and 75th percentiles, respectively; whiskers above and below the box denote the maximum and minim values. CAS: carotid artery stenosis.

The results of the simple and multiple linear regression analyses for serum omentin and vaspin levels are reported in Table 2 and Table 3, respectively. On simple linear regression analyses, increasing omentin levels were associated with increasing age, stroke severity (as reflected by NIHSSadm and NIHSSdis), history of atrial fibrillation, hypertension, congestive heart failure, history of myocardial infarction and ipsilateral carotid artery stenosis. Conversely, a negative association was observed between circulating omentin levels and TIA diagnosis. After adjustment for all previous confounders, the associations between omentin levels and NIHSSdis, history of atrial fibrillation, hypertension, congestive heart failure, history of myocardial infarction and TIA did not retain their statistical significance. Yet, the multiple linear regression analysis revealed that increasing omentin levels were independently associated with increasing age (LRC = 0.170, 95%CI: 0.063–0.277; *p* = 0.002), higher NIHSSadm (LRC = 0.290, 95%CI: 0.063–0.516; *p* = 0.013) and ipsilateral carotid artery stenosis (LRC = 3.411, 95%CI: 0.194–6.628; *p* = 0.038). Omentin also appeared strongly correlated to increasing age (Spearman rho coefficient:+0.303; *p* < 0.001) (Figure 1) and admission stroke severity (Spearman rho coefficient:+0.351; *p* < 0.001) (Figure 2). We also assessed the circulating levels of omentin in ACI patients stratified according to presence of ipsilateral carotid artery stenosis (≥50% by NASCET criteria). As shown in Figure 3, significantly higher omentin levels were measured in patients with ipsilateral carotid artery stenosis compared to patients without evidence of carotid artery disease (13.3 ± 8.9 ng/mL vs. 9.5 ± 5.5 ng/mL, *p* = 0.014). With respect to the measurements of vaspin concentrations, as shown in Table 3, the simple linear regression analyses revealed no significant associations with clinical characteristics, diagnostic results or outcomes in ACI patients (thus, no multiple linear regression analysis was performed).

## 4. Discussion

Our study showed that during the early phase of ACI, increasing omentin levels are independently associated with increasing age, higher stroke severity and ipsilateral carotid artery stenosis in patients presenting within 24 h from ACI symptom onset. With respect to outcome events during the follow-up period, no associations were detected between circulating omentin levels and one-month mortality or stroke recurrence within 90 days from the index event. Although circulating omentin did not appear associated with underlying stroke aetiologies according to TOAST classification, significantly higher omentin levels were measured in patients with moderate or severe (≥50%) ipsilateral carotid artery stenosis compared to patients without evidence of carotid artery disease. Conversely, no associations were uncovered between circulating vaspin levels and clinical characteristics, underlying stroke aetiologies, diagnostic results or clinical outcomes in ACI patients.

Our results lend support to the hypothesis that serum omentin may comprise a prognostic biomarker of stroke severity in the early phase of ACI, as indicated by the independent positive association between serum omentin concentrations and neurological impairment on admission after adjustment for potential confounders. In line with these findings, previous research in patients with coronary artery disease (CAD) has demonstrated that circulating omentin is positively correlated with the severity and extent of myocardial hypoperfusion and injury in the early phase of acute myocardial infarction (AMI) [29]. In patients with AMI, the upregulation of expression and systemic release of omentin has also been shown to confer resistance to acute myocardial ischemic damage and apoptosis in the heart [29]. Accordingly, in the setting of ACI, evidence from animal models of cerebral ischemia has indicated that circulating omentin: (1) improves the ischemic brain injury by suppressing oxidative stress, apoptotic and postischemic inflammatory cascades, (2) precipitates vasodilation and (3) promotes the postischemic revascularization in the brain by regulating the endothelial nitric oxide synthase (eNOS) and vascular endothelial growth factor (VEGF) signalling pathways [30].

It is thus unsurprising that omentin has also been proposed as a candidate biomarker for risk stratification for stroke. In a large, prospective case-cohort study comprising 2084 participants, increasing omentin concentrations were shown to be related to a significantly higher risk for stroke, with an approximately twofold increase in the likelihood of stroke per doubling omentin concentrations [15]. In the same study, a stronger association between serum omentin and the risk for stroke was observed in metabolically healthy individuals compared to those with metabolic syndrome [15]. The latter evidence suggests that (1) the presence of concomitant cardiovascular risk factors may confound the interpretation of measurements of serum adipokine levels in real-life ACI patients, and (2) circulating omentin may subserve different functions in patients with cardiovascular comorbidities compared to metabolically healthy individuals.

In fact, opposite associations between circulating omentin concentrations and the risk for cardiovascular events have been documented in population-based cohorts, in individuals with diabetes compared to nondiabetic patients [12,31,32,33]. In addition, “paradoxical” concentrations of adipokines have been noted in obese ACI patients and have been linked to the “obesity paradox”, that refers to an improved prognosis in terms of cerebrovascular disease burden, morbidity and mortality noted in obese ACI patients as compared to their normal-weight counterparts [34,35]. As circulating adipokine levels also depend on additional cardiovascular risk factors [6], comparisons of results from different studies are significantly limited by differences in clinical characteristics of included ACI patients. In contrast to our results, previous studies have reported a negative correlation between circulating omentin levels and stroke severity on admission, after excluding patients with diabetes, atrial fibrillation, CAD, AMI and congestive heart failure, as well as patients with ACI due to underlying aetiologies other than carotid artery disease [4,7,14].

Some further considerations should be taken into account when interpreting results of previous research on the diagnostic and prognostic yield of omentin in ACI patients. First, most studies so far have disregarded the temporal dynamics of omentin responses to acute ischemic and inflammatory stimuli, including patients at highly variable time points, often with delays of several days from ACI onset [4,7,14]. Second, contradictory findings on the associations between omentin and the risk of stroke have been obtained in Caucasian [15] and Asian patient populations [4,7,14], indicating that the generalizability of observed associations is likely limited by additional differences in genetic and cardiovascular risk factors in different ethnic groups [36,37]. Third, given the observed disparities in reference ranges of serum adipokines across studies, it is possible that methodological constraints exist, and standardization of assays is warranted to ensure the comparability of results between different laboratories [7,14].

In the present study, serum omentin was significantly associated with the presence of carotid artery disease in ACI patients. These results are in accordance with experimental data that implicate omentin in processes of vascular inflammation, atheroma formation and plaque ulceration and rupture [7,12,13]. Based on the current findings, we hypothesize that, during the acute phase of an ACI, a compensatory upregulation of omentin may occur in response to ischemic and inflammatory stimuli in the brain or vascular tissue [31]. Longitudinal data with serial omentin measurements are required to (1) evaluate whether elevation of omentin levels precedes or ensues ACI, (2) establish the potential causality of noted effects and (3) assess whether noted associations are retained after adjustment for baseline (i.e., prestroke) concentrations. Previous studies have highlighted that patients with higher omentin levels at baseline may have reduced risk for poor function outcome and 1-year mortality following ACI [7,14]. Thus, the absence of observed associations between circulating omentin levels with major cerebrovascular events in the present study may be attributed to the lack of adjustment for baseline (i.e., prestroke) omentin levels; although an alternative explanation is that less pronounced associations may have been missed due to the lack of refined functional outcome assessment (e.g., using modified Rankin Scale) following ACI.

Finally, with respect to vaspin measurements, we detected no associations between circulating vaspin concentrations and clinical characteristics, diagnostic results and outcomes of ACI patients. To date, however, only a limited number of proof-of-concept studies have reported associations between circulating vaspin and stroke severity or prognosis in ACI patients, while these findings could not be replicated in subsequent ACI cohorts [10,16,28,38,39]. Based on our findings, we hypothesize that, in comparison to omentin, vaspin may comprise a less sensitive biomarker for stroke. Consequently, further investigations are required to determine whether the diagnostic and prognostic utility of vaspin may be improved by combination with other biomarkers or clinical scores.

Certain limitations should be acknowledged for an accurate interpretation of our results. First, at the outset of the study, the prospective data acquisition did not entail recording of biometric measures (e.g., waist circumference), acute stroke treatments (including intravenous thrombolysis and endovascular thrombectomy) or long-term functional outcomes. Thus, analyses on the association of the previous variables with adipokine concentrations were not possible, and these should be addressed in the context of well-designed observational studies or randomized clinical trials. Second, although CTA or MRA was performed as part of the comprehensive stroke work-up, the potential presence of intracranial arteriosclerosis was not recorded systematically in the present study; however, given the findings of the present analysis, future studies should investigate the potential role of adipokines as biomarkers for the presence of intracranial arteriosclerosis. Third, due to sample size constrains, propensity score matched analyses based on concomitant cardiovascular risk factors could not be performed. Consequently, studies with larger sample sizes that will allow confirmatory statistical analyses are required to corroborate the present results. Fourth, due to additional methodological limitations, including potential confounders at baseline, type II errors cannot be excluded. However, the real-life data presented herein provide crude estimates of expected serum adipokine levels in ACI patients; thus, prospective, well-designed studies with meticulous sample size calculations are warranted to uncover potentially missed effects and establish the diagnostic and prognostic yield of novel adipokines in ACI patients.

In conclusion, the results of the present report suggest that, in the early phase of ACI, serum omentin may serve as a useful prognostic and diagnostic biomarker for stroke, which positively correlates to stroke severity and carotid artery disease in ACI patients. Conversely, the potential role of vaspin in stroke could be further elucidated in larger prospective studies, possibly in conjunction with additional biomarkers or biomarker panels, and clinical scores. Finally, the data provided herein highlight that the early kinetics of serum omentin and vaspin should be further investigated to provide insight into the pathophysiological role of novel adipokines as stroke biomarkers in real-life settings.

## Figures and Tables

**Table 1:** Baseline characteristics, neuroimaging findings, ischemic stroke classification and clinical outcomes of ACI patients.

Variables	Patients with ACI (*n* = 135)
Age (years), mean (SD)	59 ± 10
Sex	
Male, *n* (%)	92 (68)
Type of ACI	
TIA, *n* (%)	30 (22)
**Neurological deficit on admission**	
NIHSSadm, median (IQR)	3 (1–7)
**Comorbidities, *n* (%)**	
History of atrial fibrillation	15 (11)
Diabetes	38 (28)
Hypertension	70 (52)
Dyslipidaemia	76 (56)
Congestive heart failure	6 (4)
History of stroke/TIA	36 (27)
History of myocardial infarction	26 (19)
Current smoking	65 (48)
Alcohol misuse	18 (13)
Peripheral artery disease	3 (2)
**TOAST classification**	
Large-artery atherosclerosis, *n* (%)	28 (21)
Cardioembolism, *n* (%)	35 (26)
Small-vessel occlusion, *n* (%)	6 (4)
Stroke of other determined aetiology, *n* (%)	12 (9)
Stroke of undetermined aetiology, *n* (%)	54 (40)
	
Ipsilateral carotid artery stenosis ≥ 50%, *n* (%)	18 (13)
Contralateral carotid artery stenosis ≥ 50%, *n* (%)	15 (11)
Vertebral artery stenosis ≥ 50%, *n* (%)	3 (2)
Left atrial enlargement moderate or severe, *n* (%)	12 (9)
Valvular disease, *n* (%)	7 (5)
Atrial fibrillation on Holter monitor, *n* (%)	23 (17)
CRP mg/L, mean (SD)	8.2 ± 10.9
Omentin ng/mL, mean (SD)	10.2 ± 6.3
Vaspin ng/mL, mean (SD)	146.6 ± 177.1
	
Early neurological deterioration, *n* (%)	8 (6)
NIHSSdis median (IQR)	1 (0–3)
Recurrent stroke within 3 months, *n* (%)	7 (5)
Death within 3 months, *n* (%)	6 (4)

ACI: acute cerebral infarction, SD: standard deviation, TIA: transient ischemic attack, IQR: interquartile range, NIHSS_adm_/NIHSS_dis_: National Institutes of Health Stroke Scale on admission/at discharge, TOAST: Trial of Org 10172 in Acute Stroke Treatment, CRP: c-reactive protein.

**Table 2:** Simple and multiple linear regression analyses on the associations of omentin levels with baseline characteristics, neuroimaging findings and outcomes in ACI patients.

	Simple Linear Regression			Multiple Linear Regression		
Variable	LRC	95% CI	*p*-value	LRC	95% CI	*p*-value
Age	0.175	0.075, 0.274	**0.001**	0.170	0.063, 0.277	0.002
Male sex	0.786	−1.513, 3.085	0.500	-		-
NIHSSadm	0.325	0.141, 0.509	**0.001**	0.290	0.063, 0.516	0.013
TIA	−0.285	−5.459, −0.237	**0.033**	−0.017	−3.668, 1.634	0.449
History of atrial fibrillation	5.261	1.865, 8.658	**0.033**	3.72	−0.030, 7.471	0.052
Diabetes	0.801	−1.551, 3.153	0.501	-		-
Hypertension	2.507	0.425, 4.588	**0.019**	0.968	−1.349, 3.286	0.409
Dyslipidaemia	−1.614	−3.737, 0.501	0.135	-		-
Congestive heart failure	5.980	0.519, 11.439	**0.032**	−1.771	−8.728, 5.186	0.615
History of stroke/TIA	0.080	−2.329, 2.450	0.947	-		-
History of myocardial infarction	3.941	1.320, 6.561	**0.004**	2.157	−0.875, 5.189	0.161
Current smoking	0.464	−1.234, 2.161	0.590	-		-
Alcohol misuse	1.484	−1.672, 4.641	0.354	-		-
Peripheral artery disease	0.612	−6.523, 7.747	0.865	-		-
						
Ipsilateral carotid artery stenosis ≥ 50%	3.905	0.813, 6.997	**0.014**	3.411	0.194, 6.628	0.038
Contralateral carotid artery stenosis ≥ 50%	1.716	−1.715, 5.147	0.324	-		-
Vertebral artery stenosis ≥ 50%	−3.582	−10.660, 3.500	0.319	-		-
Left atrial enlargement	3.108	−0.235, 6.452	0.068	2.844	−0.375, 6.063	0.082
Valvular disease	−4.811	−11.946, 2.324	0.183	-		-
Atrial fibrillation on Holter monitor	2.483	−0.242, 5.209	0.074	1.046	−1.788, 3.881	0.465
CRP	−0.031	−0.167, 0.105	0.647	-		-
						
						
Early neurological deterioration	0.677	−3.763, 5.118	0.763	-		-
NIHSSdis	0.307	0.035, 0.580	**0.027**	−0.387	−0.813, 0.04	0.075
Recurrent stroke within 3 months	−0.771	−5.898, 4.357	0.767	-		-
Death within 3 months	1.759	−4.757, 8.276	0.594	-		-

ACI: acute cerebral infarction, LRC: linear regression coefficient, CI: confidence interval, IQR: interquartile range, TIA: transient ischemic attack, NIHSS_adm_/NIHSS_dis_: National Institutes of Health Stroke Scale on admission/at discharge, TOAST: Trial of Org 10172 in Acute Stroke Treatment. Statistically significant results (*p* < 0.05) are highlighted in bold. All associations in linear regression models are reported using unstandardized linear regression coefficients (LRC), with their corresponding 95% confidence intervals (95%CI).

**Table 3:** Simple linear regression analyses on the associations of vaspin levels with baseline characteristics, neuroimaging findings and outcomes in ACI patients.

Simple Linear Regression			
Variable	LRC	95% CI	*p*-Value
Age	1.721	−1.400, 4.842	0.277
Male sex	19.442	−50.072, 88.956	0.581
NIHSSadm	−0.308	−6.952, 6.337	0.927
TIA	−15.700	−85.749, 54.350	0.658
History of atrial fibrillation	9.640	−97.736, 117.016	0.859
Diabetes	−18.963	−90.150, 52.223	0.599
Hypertension	39.458	−25.330, 104.250	0.230
Dyslipidaemia	58.887	−6.492, 124.267	0.077
Congestive heart failure	23.013	−158.334, 204.361	0.802
History of stroke/TIA	−24.251	−96.379, 47.877	0.507
History of myocardial infarction	53.163	−30.235, 136.561	0.209
Current smoking	4.246	−50.447, 58.939	0.878
Alcohol misuse	−5.309	−103.451, 92.832	0.915
Peripheral artery disease	−35.744	−244.056, 172.566	0.735
Ipsilateral carotid artery stenosis ≥ 50%	−41.671	−142.947, 59.605	0.417
Contralateral carotid artery stenosis ≥50%	8.704	−99.933, 117.341	0.874
History of vertebral artery stenosis ≥ 50%	119.294	−133.935, 372.522	0.353
Left atrial enlargement	−20.432	−123.491, 82.626	0.695
Valvular disease	−78.747	−263.383, 105.888	0.387
Atrial fibrillation on Holter monitor	−55.052	−164.022, 53.919	0.318
CRP	0.3694	−3.657, 4.395	0.855
Early neurological deterioration	−50.136	−198.607, 98.334	0.505
NIHSSdis	−9.122	−19.371, 1.126	0.080
Recurrent stroke within 3 months	−19.876	−169.624, 129.873	0.793
Death within 3 months	−130.753	−353.731, 92.225	0.247

ACI: acute cerebral infarction, LRC: linear regression coefficient, CI: confidence interval, IQR: interquartile range, TIA: transient ischemic attack, NIHSSadm/NIHSSdis: National Institutes of Health Stroke Scale on admission/at discharge, TOAST: Trial of Org 10172 in Acute Stroke Treatment. There were no statistically significant results (*p* < 0.05). All associations in linear regression models are reported using unstandardized linear regression coefficients (LRC), with their corresponding 95% confidence intervals (95%CI).

## Data Availability

The data that support the findings of this study are available from the corresponding author (G.T.), upon reasonable request.
